# Spectral shifted Chebyshev collocation technique with residual power series algorithm for time fractional problems

**DOI:** 10.1038/s41598-024-58493-x

**Published:** 2024-04-15

**Authors:** Saad. Z. Rida, Anas. A. M. Arafa, Hussein. S. Hussein, Ismail G. Ameen, Marwa. M. M. Mostafa

**Affiliations:** 1 Department of Mathematics, Faculty of Science, South Valley University, Qena, 83523 Egypt; 2 Department of Mathematics, College of Science, Qassim University, Buraydah, Saudi Arabia; 3 Department of Mathematics and Computer Science, Faculty of Science, Port Said University, Port Said, Egypt

**Keywords:** Shifted Chebyshev polynomials of the second kind, Residual power series algorithm, Fractional derivatives, Hyperbolic equation with time fractional, Time fractional pseudo hyperbolic equations, Numerical results., Mathematics and computing, Pure mathematics

## Abstract

In this paper, two problems involving nonlinear time fractional hyperbolic partial differential equations (PDEs) and time fractional pseudo hyperbolic PDEs with nonlocal conditions are presented. Collocation technique for shifted Chebyshev of the second kind with residual power series algorithm (CTSCSK-RPSA) is the main method for solving these problems. Moreover, error analysis theory is provided in detail. Numerical solutions provided using CTSCSK-RPSA are compared with existing techniques in literature. CTSCSK-RPSA is accurate, simple and convenient method for obtaining solutions of linear and nonlinear physical and engineering problems.

## Introduction

Mathematical modeling of various nonlinear phenomena, which can be expressed using nonlinear differential equations (DEs) is more complex and difficult than modeling linear phenomena. Such phenomena have an important role in the study of many scientific fields and often described by ordinary differential equations (ODEs) and PDEs. Although solving PDEs is more difficult than solving ODEs, these equations are widely used in physics and mathematical problems. The fractional arrangement has been used to generalize these equations by researchers in recent decades, and these equations have been known as fractional partial differential equations (FPDEs). It is difficult to obtain accurate solutions to such equations in their nonlinear state. In the literature, several mathematical methods are presented for solving these equations as Adomian decomposition method (ADM)^1,2^, variational iteration method (VIM)^3^, Iterative Laplace transform method^4^, Sumudu transform method^5^, finite difference method^6^, Tau method^7^, homotopy perturbation method (HPM)^8^, wavelet methods^9^, homotopy analysis method^10^, variational homotopy perturbation iteration method (VHPIM)^11^, finite element method^12^, modified HPM^13^ and Jacobi collocation^14^.

RPSA is an efficient, powerful and simple technique to create a power series solution that can be handled without discretization, linearization, and perturbation for linear and nonlinear equations. RPSA does not need any changes while transforming from lower to higher order. Hence, the technique can be utilized directly for problem by choosing suitable preliminary guess approximation. Researchers have used RPSA for solving different types of models, such as fuzzy differential equations^15^, fractional Burger types equations^16^, fractional gas dynamic equations^17^, KdV-Burgers equation^18^, Whitham–Broer–Kaup equations^19^, fractional time Cahn–Hilliard, Gardner equations^20,21^, Swift–Hohenberg equation^22^, fractional diffusion equation^23^, Burgers–Huxley equations^24^, Navier–Stokes equations^25^ and Lane–Emden equations^26^.

Hyperbolic PDEs is a type of more significance nonlinear models in physics of mathematical. In the last few years, there exist analytical and numerical methods to solve these problems^27,28^. In Ref.^8^ Odibat and Momani obtained the analytic and approximate solution for hyperbolic PDEs by using VIM and ADM. Khalid et al.^29^ constructed an efficient schemes called Perturbation iteration algorithm (PIA) to get approximate solutions for hyperbolic PDEs. Das and Gupta^30^ employed HAM for obtaining the approximate solution for nonlinear hyperbolic PDEs of fractional order. Pseudo-hyperbolic equations is type of high order PDEs with combination of partial derivatives concerning space and time, which describes various phenomena of physical including, diffusion of reaction, vibrations of longitudinal and physics of plasma^31,32^. In recent years, researchers and scientists have presented the numerical and analytical methods to solve the pseudo hyperbolic equation^33–35^. In Refs.^32,36^, the authors studied uniqueness, existence and stability analysis of numerical solutions for pseudo hyperbolic PDES.

The fundamental target of this study is to employ an approximate solution for time fractional hyperbolic PDEs and time fractional pseudo hyperbolic PDEs with nonlocal conditions. The method of solution is to apply properties of shifted Chebyshev polynomials of second kind (SCPSK) to reduce space hyperbolic PDEs and pseudo hyperbolic PDEs with nonlocal conditions into system of fractional ODEs, these FODEs system have been solved by employing RPSA.

The outline work is prepared as: The main definitions of Caputo fractional derivative (CFD) and fractional power series (FPS) are given in Section “Preliminaries”. Some characteristics for Chebyshev polynomials of the second kind (CPSK) are presented in Section “General characteristics of spectral Chebyshev polynomials”. The theorem utilized to discuss the method’s error analysis is presented in Section “Error analysis”. The methodology has been applied to two applications in Section “Applications of methodology”. Numerical solutions and simulations to show CTSCSK-RPSA efficiency are presented in Section “Numerical simulation”. In Section “Conclusion”, a final conclusion is drawn.

## Preliminaries

In this section, we give some essential definitions of CFD and FPS.

### Definition 1

^37–39^ The CFD of order $\beta$ for a function $\varTheta (t)\in \texttt{C}_{q}$, $q \geq -1$ is defined as belows:

1 $$\begin{aligned} {\mathfrak {D}}^\beta \varTheta (t)={\mathfrak {I}}^{m-\beta } {\mathfrak {D}}^m \varTheta (t)= \frac{1}{\varGamma (m-\beta )}\displaystyle \int _{0}^{t} (t-\nu )^{m-\beta -1} {\mathfrak {D}}^{m}\varTheta (\nu )d\nu , \ t>0,\ m-1<\beta < m. \end{aligned}$$

### Definition 2

^37–39^ The Caputo fractional partial derivative (CFPD) of order $\beta$ for a function $\varTheta (x,t)\in \texttt{C}_{q}$, $q \geq -1$ is given by:

2 $${\mathfrak{D}}_{t}^{\beta } \varTheta(x,t) = \left\{ {\begin{array}{*{20}l} {\frac{1}{{\varGamma(m - \beta )}}\int\limits_{0}^{t} {(t - \nu )^{{m - \beta - 1}} } \;\frac{{\partial ^{m} \varTheta(x,\nu )}}{{\partial \nu ^{m} }}d\nu ,} \hfill & {m - 1 < \beta < m,} \hfill \\ {\frac{{\partial ^{m} \varTheta(x,t)}}{{\partial t^{m} }},} \hfill & {\beta = m \in \mathbb{N}.} \hfill \\ \end{array} } \right.$$

The CFD satisfies linear property similar to integer order differentiation:

$$\begin{aligned} \quad {\mathfrak {D}}^\beta \left[ \lambda _1 \varTheta _1(t)+\lambda _2 \varTheta _2(t)+ \cdots +\lambda _m \varTheta _m(t) \right] =\left[ \lambda _1 {\mathfrak {D}}^\beta \varTheta _1(t)+\lambda _2 {\mathfrak {D}}^\beta \varTheta _2(t)+ \cdots +\lambda _m {\mathfrak {D}}^\beta \varTheta _m(t) \right] , \end{aligned}$$

where $\lambda _1, \lambda _2, \ldots , \lambda _m$ are constants.

The major properties for the Caputo derivative are:

3 $$\begin{aligned} {\mathfrak {D}}^\beta {\textbf{k}}=0, \ {\textbf{k}}\quad is \ constant. \end{aligned}$$

4 $${\mathfrak{D}}^{\beta } t^{\upsilon } = \left\{ {\begin{array}{*{20}l} {\frac{{\varGamma(\upsilon + 1)}}{{\varGamma(\upsilon + 1 - \beta )}}t^{{\upsilon - \beta }} ,} \hfill & {for\;\upsilon \in {\mathcal{N}}_{0} ,\upsilon \ge \left\lceil \beta \right\rceil ,} \hfill \\ {0,} \hfill & {for\;\upsilon \in {\mathcal{N}}_{0} ,\upsilon < \left\lceil \beta \right\rceil ,} \hfill \\ \end{array} } \right.$$

where $\lceil \beta \rceil$ denote to the smallest integer greater than or equal to $\beta$, where ${\mathcal {N}}_{0}= \{0,1,2, \ldots \}$.

### Definition 3

^40,41^ The power series which has the formula

$$\begin{aligned} \sum _{l=0}^\infty \ \vartheta _{ l}(\tau -\tau _0) ^{l\beta }=\vartheta _{ 0}+\vartheta _{ 1}(\tau -\tau _0) ^{\beta }+\vartheta _{ 2}(\tau -\tau _0) ^{2\beta }+ \cdots ,0\le l-1<\beta \le l,\ l \in {\mathbb {N}}, \ and\ \tau \ge \tau _0, \end{aligned}$$

is called FPS about $\tau _0$.

There exist the three possibilities for convergence of the FPS $\sum _{l=0}^\infty \ \vartheta _{ l}(\tau -\tau _0) ^{l\beta }$, which are:

- The series converges only for $\tau = \tau _0$, that is, the radius of convergence equal zero.
- The series converges for all $\tau \ge \tau _0$, that is, the radius of convergence equal $\infty$.
- The series converges for $\tau _0\le \tau <\tau _0+{\mathcal {R}}$, for some positive real number ${\mathcal {R}}$ and diverges for $\tau >\tau _0 +{\mathcal {R}}$ , where ${\mathcal {R}}$ is the radius of convergence for the FPS.

### Definition 4

^40^ The multiple FPS at $\tau =\tau _0$ is defined as:

$$\begin{aligned} \sum _{r=0}^\infty \sum _{j=0}^{l-1}\ P_{r j}(x)(\tau -\tau _0) ^{r\beta +j}, 0\le l-1<\beta \le l, l \in {\mathbb {N}}\ and \ \tau _0\le \tau \le \tau _0+{\mathcal {R}}. \end{aligned}$$

## General characteristics of spectral Chebyshev polynomials

We recall some main expressions of spectral SCPSK that are utilized in this paper.

### Definition 5

^42^ The spectral CPSK ${\mathfrak {T}} _m({\textbf{s}})$ over the interval $[-1,1]$ can be defined as:

$$\begin{aligned} {\mathfrak {T}}_m({\textbf{s}})=\dfrac{sin(m+1)\xi }{sin(\xi )}, \end{aligned}$$

where $${\textbf{s}}=cos(\xi ),$$ $$\xi \in [0,\pi ].$$

The orthogonality formula of CPSK with respect to weight function $\omega ({\textbf{s}})=\sqrt{1-{\textbf{s}}^2}$ as:

$$\begin{aligned} <{\mathfrak {T}} _m({\textbf{s}}),{\mathfrak {T}} _j ({\textbf{s}})>=\displaystyle \int _{-1}^{1} \omega ({\textbf{s}})\ {\mathfrak {T}} _m({\textbf{s}}){\mathfrak {T}} _j ({\textbf{s}}) d{\textbf{s}}={\left\{ \begin{array}{ll} 0 &{} \text {if } m\ne j,\\ \frac{\pi }{2} &{} \text {if } m= j.\\ \end{array}\right. } \end{aligned}$$

The recurrence form of polynomials ${\mathfrak {T}} _m({\textbf{s}})$ can be written as:

$$\begin{aligned} {\mathfrak {T}} _m({\textbf{s}})=2{\textbf{s}}{\mathfrak {T}} _{m-1}({\textbf{s}})-{\mathfrak {T}} _{m-2}({\textbf{s}}), \ m=2,3,4, \ldots , \end{aligned}$$

where

$$\begin{aligned} {\mathfrak {T}} _0({\textbf{s}})=1, \ {\mathfrak {T}} _1({\textbf{s}})=2{\textbf{s}}. \end{aligned}$$

The explicit formula of ${\mathfrak {T}} _m({\textbf{s}})$ as:

5 $$\begin{aligned} {\mathfrak {T}} _m({\textbf{s}})=\sum _{i=0}^{\lceil \frac{m}{2}\rceil } (-1)^{i}\frac{2^{m-2i}\varGamma (m-i+1)}{\varGamma {(m-2i+1)\varGamma (i+1)}}{\textbf{s}}^{m-2i},\ m >0, \end{aligned}$$

where $\lceil \frac{m}{2}\rceil$ denotes the integral part of $\frac{m}{2}$.

### Definition 6

^42^ The SCPSK ${\mathfrak {T}}^{*} _m(x)$ is defined on [0, 1] as:

$$\begin{aligned} {\mathfrak {T}}^{*} _m(x)={\mathfrak {T}}_{m}(2x-1). \end{aligned}$$

The orthogonal property of SCPSK with respect to weight function $\omega ^{*}(x)=\sqrt{x-x^2}$ is given as below:

$$\begin{aligned} <{\mathfrak {T}}^{*} _m(x),{\mathfrak {T}}^{*} _j(x)>=\displaystyle \int _{0}^{1} \sqrt{x-x^2}\ {\mathfrak {T}}^{*} _m(x){\mathfrak {T}}^{*} _j(x) dx={\left\{ \begin{array}{ll} 0 &{} \text {if } m\ne j,\\ \frac{\pi }{8} &{} \text {if } m= j,\\ \end{array}\right. } \end{aligned}$$

The recurrence relation of SCPSK:

$$\begin{aligned} {\mathfrak {T}}^{*}_m(x)=2(2x-1){\mathfrak {T}}^{*}_{m-1}(x)-{\mathfrak {T}}^{*}_{m-2}(x), \ m=2,3,4,\ldots \end{aligned}$$

where

$$\begin{aligned} {\mathfrak {T}}^{*}_0(x)=1, \ {\mathfrak {T}}^{*}_1(x)=4x-2. \end{aligned}$$

The analytical expressions of SCPSK ${\mathfrak {T}}^{*}_m(x)$ of degree *m* can be given as:

6 $$\begin{aligned} {\mathfrak {T}}^{*}_m(x)=\sum _{i=0}^m (-1)^{i}\frac{2^{2m-2i}\varGamma (2m-i+2)}{\varGamma {(2m-2i+1)\varGamma (i+1)}}x^{m-i},\ m >0. \end{aligned}$$

The function $u(x)\in {\mathfrak {L}}_{2}[0,1]$ can be defined by SCPSK ${\mathfrak {T}}^{*}_i(x)$ as follows:

7 $$\begin{aligned} u(x)=\sum _{i=0}^\infty \vartheta _{i} {\mathfrak {T}}^{*}_i(x), \end{aligned}$$

where the coefficients $\vartheta _{i}$ are given by:

8 $$\begin{aligned} \vartheta _{i}=\frac{8}{\pi } \int _{0}^{1} \sqrt{x-x^2}\ u(x) {\mathfrak {T}}^{*}_i(x)dx, i=0,1,2,\dots \end{aligned}$$

In practice, we truncate the infinite series up to $(n+1)$ terms of SCPSK as follows:

9 $$\begin{aligned} u_n(x) =\sum _{i=0}^n \vartheta _{i}{\mathfrak {T}}^{*}_i(x). \end{aligned}$$

### Theorem 1

Assume that $u_n(x)$ be series approximation of spectral SCPSK defined by Eq. (9), then ${\mathfrak {D}}^{\beta }u_n (x)$ is given as:

10 $$\begin{aligned} {\mathfrak {D}}^{\beta }u_n (x)=\sum _{i=\lceil \beta \rceil }^n \sum _{k=0}^{i-\lceil \beta \rceil } \vartheta _{i}\ \varOmega _{i,k} ^{(\beta )}\ x^{i-k-\beta }, \end{aligned}$$

where $\varOmega _{i,k} ^{(\beta )}$ is defined as:

$$\begin{aligned} \varOmega _{i,k} ^{(\beta )}= (-1)^{k}\frac{\ 2^{2i-2k} \varGamma (2i-k+2) \ \varGamma (i-k+1)}{\varGamma (k+1) \varGamma (2i-2k+2) \varGamma (i-k-\beta +1)}. \end{aligned}$$

### Proof

(see Ref.^42^). $$\square$$

## Error analysis

In this section, the following theorem proves an error analysis of the method.

### Theorem 2

Suppose a function $\varPhi (x) \in [0,1]$ which is continuous and differentiable up to $(n + 1)$ times. Let   $u_n(x) =\displaystyle \sum _{i=0}^n \vartheta _{i}{\mathfrak {T}}^{*}_i(x)$ be the best square approximation function of $\varPhi (x)$, then

11 $$\begin{aligned} \Vert \varPhi (x)- u_n(x) \Vert \le \dfrac{{\mathbb {M}} ({\mathcal {K}}^{n+1}) }{2 \ \varGamma (n+2)}\sqrt{\dfrac{\pi }{2}}, \end{aligned}$$

where $${\mathbb {M}}=\max _{x\in [0,1]}\ \varPhi ^{(n+1)}(x)$$ and $${\mathcal {K}}= \max \{x_0,x-x_0\}$$.

### Proof

We approximate function $\varPhi (x)$ by Taylor series as:

12 $$\begin{aligned} \varPhi (x)=\varPhi (x_0)+\varPhi '(x_0)\frac{(x-x_0)}{\varGamma (2)}+\varPhi ''(x_0)\dfrac{(x-x_0)^2}{\varGamma (3)}+\cdots +\varPhi ^{(n)}(x_0)\dfrac{(x-x_0)^{n}}{\varGamma (n+1)}+\varPhi ^{(n+1)}(\zeta ) \dfrac{(x-x_0)^{n+1}}{\varGamma (n+2)}, \end{aligned}$$

where $$x_0\in [0,1]$$ and $$\zeta \in [x_0,x]$$.

Let

13 $$\begin{aligned} y_n(x)=\varPhi (x_0)+\varPhi '(x_0)\frac{(x-x_0)}{\varGamma (2)}+\varPhi ''(x_0)\dfrac{(x-x_0)^2}{\varGamma (3)}+\cdots +\varPhi ^{(n)}(x_0)\dfrac{(x-x_0)^{n}}{\varGamma (n+1)}, \end{aligned}$$

then

14 $$\left\| {\varPhi(x) - y_{n} (x)} \right\| = \left| {\varPhi^{{(n + 1)}} (\zeta )\frac{{(x - x_{0} )^{{n + 1}} }}{{\varGamma(n + 2)}}} \right|.$$

Since $u_n(x) = {\mathop {\sum }\limits _{i=0}^n} {{\vartheta _{i}}^{*}_{i(x)}}$, is the best square approximation function of $\varPhi (x)$, we have

$$\begin{aligned} \left\| {\varPhi (x) - u_{n} (x)} \right\|^{2} & \le \left\| {\varPhi (x) - y_{n} (x)} \right\|^{2} = \int\limits_{0}^{1} {w^{*} (x)\left( {\varPhi (x) - y_{n} (x)} \right)^{2} } dx \\ & = \int\limits_{0}^{1} {w^{*} } (x)\left( {\varPhi ^{{(n + 1)}} (\zeta )\frac{{(x - x_{0} )^{{n + 1}} }}{{\varGamma (n + 2)}}} \right)^{2} dx \\ & \le \frac{{{\mathbb{M}}^{2} }}{{(\varGamma (n + 2))^{2} }}\int\limits_{0}^{1} {\sqrt {x - x^{2} } } \left( {(x - x_{0} )^{{n + 1}} } \right)^{2} dx. \\ \end{aligned}$$

Hence ${\mathcal {K}}=\max \{x_0,x-x_0\}$, we get

15 $$\begin{aligned} \left\| {\varPhi (x) - u_{n} (x)} \right\|^{2} & \le \frac{{{\mathbb{M}}^{2} {\mathcal{K}}^{{(2n + 2)}} }}{{(\varGamma (n + 2))^{2} }}\int_{0}^{1} {\sqrt {x - x^{2} } } dx \\ & = \frac{{{\mathbb{M}}^{2} {\mathcal{K}}^{{(2n + 2)}} }}{{(\varGamma (n + 2))^{2} }}\frac{\pi }{8}. \\ \end{aligned}$$

By taking square root of both sides for Eq. (15), we get

16 $$\begin{aligned} \begin{aligned} \Vert \varPhi (x)-u_n(x) \Vert \le \dfrac{{\mathbb {M}} ({\mathcal {K}}^{n+1}) }{2\ \varGamma (n+2)}\sqrt{\dfrac{\pi }{2}}. \end{aligned} \end{aligned}$$

$$\square$$

## Applications of methodology

The principal objective of this section is to obtain an approximate solution for time fractional hyperbolic PDEs and time fractional pseudo hyperbolic PDEs with nonlocal conditions.

- Time fractional hyperbolic PDEs^29^
  17 $$\begin{aligned} {\mathfrak {D}}^{\beta }_{t} \varTheta (x,t)-\mu \ {\mathfrak {D}}^{2}_{x} \varTheta (x,t)- {\mathbb {L}}\left( \varTheta (x,t)\right) =0, \ 1<\beta \le 2,\ x\in [0,X],\ t >0, \end{aligned}$$
  with subject to initial conditions (ICs) and boundary conditions (BCs):
  18 $$\begin{aligned} {\left\{ \begin{array}{ll} \varTheta (x,0)=f_1(x), &{} {\mathfrak {D}}_{t} \varTheta (x,0)=f_2(x),\\ \varTheta (0,t)= A_1(t), &{} \varTheta (X,t)=A_2(t), \\ \end{array}\right. } \end{aligned}$$
  where $\mu \in {\mathbb {R}}$ and ${\mathbb {L}}$ is non linear operator. Assume $\varTheta _n(x,t)$ is approximated as:
  19 $$\begin{aligned} \varTheta _n(x,t)=\sum _{i=0}^n \vartheta _{i}(t){\mathfrak {T}}^{*}_i(x). \end{aligned}$$
  Let us to utilize the approximation of $\varTheta _n(x,t)$ which is defined in Eq. (19) as following steps:
  Step (I) By applying Theorem (1) and Eqs. (17) and (19), we have
    20 $$\begin{aligned} \sum _{i=0}^n {\mathfrak {D}}^{\beta }_{t} \vartheta _{i}(t){\mathfrak {T}}^{*}_i(x)-\mu \sum _{i=\lceil \beta \rceil }^n \sum _{k=0}^{i-\lceil \beta \rceil } \vartheta _{i}(t) \varOmega _{i,k} ^{(\beta )} x^{i-k-\beta }-{\mathbb {L}}\left( \sum _{i=0}^n \vartheta _{i}(t){\mathfrak {T}}^{*}_i(x)\right) =0. \end{aligned}$$
  Step (II) Now we collocate Eq. (20) at $x_p,$ $p = 0,1,2, ldots n-\lceil \beta \rceil$ and the collocation point of SCPSK ${\mathfrak {T}}^{*}_{n+1-\lceil \beta \rceil } (x)$, we have a system of fractional order differential equations (FODEs) as:
    21 $$\begin{aligned} \sum _{i=0}^n {\mathfrak {D}}^{\beta }_{t} \vartheta _{i}(t){\mathfrak {T}}^{*}_i(x_p)-\mu \sum _{i=\lceil \beta \rceil }^n \sum _{k=0}^{i-\lceil \beta \rceil } \vartheta _{i}(t) \varOmega _{i,k} ^{(\beta )} x_p^{i-k-\beta }-{\mathbb {L}}\bigg (\sum _{i=0}^n \vartheta _{i}(t){\mathfrak {T}}^{*}_i(x_p)\bigg )=0. \end{aligned}$$
  Step (III) Substituting Eq. (19) into Eqs. (18), we can obtain $(\lceil \beta \rceil +1)$ algebraic equations as:
    22 $$\left\{ {\begin{array}{*{20}l} {{\text{ }}\sum\limits_{{i = 0}}^{n} {\vartheta _{i} } (0){\mathfrak{T}}_{i}^{*} (x) = f_{1} (x),} \hfill \\ {\sum\limits_{{i = 0}}^{n} {{\mathfrak{D}}_{t} } \vartheta _{i} (0){\mathfrak{T}}_{i}^{*} (x) = f_{2} (x),} \hfill \\ \end{array} } \right.$$
    where BCs
    23 $$\left\{ {\begin{array}{*{20}l} {{\text{ }}\sum\limits_{{i = 0}}^{n} {\vartheta _{i} } (t){\mathfrak{T}}_{i}^{*} (0) = A_{1} (t),} \hfill \\ {\sum\limits_{{i = 0}}^{n} {\vartheta _{i} } (t){\mathfrak{T}}_{i}^{*} (X) = A_{2} (t).} \hfill \\ \end{array} } \right.$$
  To obtain the unknown coefficients $\vartheta _0(t),\vartheta _1(t),\vartheta _2(t), \ldots ,\vartheta _n(t)$, combing Eqs. (21)–(23), we have system of FODEs, which can be solved by utilizing RPSA. To determine the unknown coefficients of $\vartheta _0(t),\vartheta _1(t),\vartheta _2(t), \ldots ,\vartheta _n(t)$, we take $n=2$ and $n=3$ in Eq. (21), respectively:
  24 $$\begin{aligned} {\left\{ \begin{array}{ll} {\mathfrak {D}}^{\beta }_{t} \vartheta _{0}(t)- {\mathfrak {D}}^{\beta }_{t} \vartheta _{2}(t)-32\mu \vartheta _{2}(t)-{\mathbb {L}}\bigg (\vartheta _{0}(t)-\vartheta _{2}(t)\bigg )=0. \end{array}\right. } \end{aligned}$$
  25 $$\begin{aligned} {\left\{ \begin{array}{ll} \begin{aligned} {\mathfrak {D}}^{\beta }_{t} \vartheta _{0}(t)-{\mathfrak {D}}^{\beta }_{t} \vartheta _{1}(t)+ {\mathfrak {D}}^{\beta }_{t} \vartheta _{3}(t)-\mu \big (32\vartheta _{2}(t)-96\vartheta _{3}(t)\big )-{\mathbb {L}}\bigg (\vartheta _{0}(t) -\vartheta _{1}(t)+\vartheta _{3}(t)\bigg )=0,\\ {\mathfrak {D}}^{\beta }_{t} \vartheta _{0}(t)+{\mathfrak {D}}^{\beta }_{t} \vartheta _{1}(t)- {\mathfrak {D}}^{\beta }_{t} \vartheta _{3}(t)-\mu \big (32\vartheta _{2}(t)+96\vartheta _{3}(t)\big )-{\mathbb {L}}\bigg (\vartheta _{0}(t) +\vartheta _{1}(t)-\vartheta _{3}(t)\bigg )=0 \end{aligned} \end{array}\right. } \end{aligned}$$
  By solving Eq. (25), we get
  26 $$\left\{ {\begin{array}{*{20}l} {{\text{ }}{\mathfrak{D}}_{t}^{\beta } \vartheta _{0} (t) - 32\mu \vartheta _{2} (t) - \frac{1}{2}[{\mathbb{L}}(\vartheta _{0} (t) - \vartheta _{1} (t) + \vartheta _{3} (t)) + {\mathbb{L}}(\vartheta _{0} (t) + \vartheta _{1} (t) - \vartheta _{3} (t))] = 0,} \hfill \\ {{\mathfrak{D}}_{t}^{\beta } \vartheta _{1} (t) - {\mathfrak{D}}_{t}^{\beta } \vartheta _{3} (t) - 96\mu \vartheta _{3} (t) + \frac{1}{2}[{\mathbb{L}}(\vartheta _{0} (t) - \vartheta _{1} (t) + \vartheta _{3} (t)) - {\mathbb{L}}(\vartheta _{0} (t) + \vartheta _{1} (t) - \vartheta _{3} (t))] = 0.} \hfill \\ \end{array} } \right.$$
  By solving Eq. (23) at $n=2$ and $n=3$, respectively. Then we get
  27 $$\left\{ {\begin{array}{*{20}l} {\vartheta _{1} (t) = \frac{1}{4}(A_{2} (t) - A_{1} (t)),} \hfill \\ {\vartheta _{2} (t) = \frac{1}{6}(A_{1} (t) + A_{2} (t)) - \frac{1}{3}\vartheta _{0} (t).} \hfill \\ \end{array} } \right.$$
  28 $$\left\{ {\begin{array}{*{20}l} {{\text{ }}\vartheta _{2} (t) = \frac{1}{6}(A_{1} (t) + A_{2} (t)) - \frac{1}{3}\vartheta _{0} (t),} \hfill \\ {\vartheta _{3} (t) = \frac{1}{8}(A_{2} (t) - A_{1} (t)) - \frac{1}{2}\vartheta _{1} (t).} \hfill \\ \end{array} } \right.$$
  By substituting Eqs. (27) and (28) into Eqs. (24) and (26), then
  29 $$\left\{ {\begin{array}{*{20}l} {{\mathfrak{D}}_{t}^{\beta } \vartheta _{0} (t) - \frac{1}{8}{\mathfrak{D}}_{t}^{\beta } (A_{1} (t) + A_{2} (t)) - 4\mu (A_{1} (t) + A_{2} (t))} \hfill \\ {\quad + 8\mu \vartheta _{0} (t) - \frac{3}{4}{\mathbb{L}}\left( {\frac{4}{3}\;\vartheta _{0} (t) - \frac{1}{6}[A_{1} (t) + A_{2} (t)]} \right),} \hfill \\ \end{array} } \right.$$
  30 $$\left\{ {\begin{array}{*{20}l} {{\mathfrak{D}}_{t}^{\beta } \vartheta _{0} (t) - \frac{{16\mu }}{3}[A_{1} (t) + A_{2} (t)] + \frac{{32\mu }}{3}\vartheta _{0} (t)} \hfill \\ {\quad - \frac{1}{2}[{\mathbb{L}}(\vartheta _{0} (t) - \frac{3}{2}\vartheta _{1} (t) + \frac{1}{8}[A_{2} (t) - A_{1} (t)])} \hfill \\ {\quad + {\mathbb{L}}(\vartheta _{0} (t) + \frac{3}{2}\vartheta _{1} (t) - \frac{1}{8}[A_{2} (t) - A_{1} (t)])],} \hfill \\ \end{array} } \right.$$
  31 $$\left\{ {\begin{array}{*{20}l} {{\mathfrak{D}}_{t}^{\beta } \vartheta _{1} (t) - \frac{1}{{12}}{\mathfrak{D}}_{t}^{\beta } (A_{2} (t) - A_{1} (t)) - 8\mu [A_{2} (t) - A_{1} (t)]} \hfill \\ {\quad + 32\mu \vartheta _{1} (t) + \frac{1}{3}[{\mathbb{L}}(\vartheta _{0} (t) - \frac{3}{2}\vartheta _{1} (t) + \frac{1}{8}[A_{2} (t) - A_{1} (t)])} \hfill \\ {\quad - {\mathbb{L}}(\vartheta _{0} (t) + \frac{3}{2}\vartheta _{1} (t) - \frac{1}{8}[A_{2} (t) - A_{1} (t)])] = 0.} \hfill \\ \end{array} } \right.$$
  RPSA assumes the solution of Eq. (29) using FPS at $t_0=0$ as:
  32 $$\begin{aligned} \vartheta _{0}(t)=\varUpsilon _{0}+\varUpsilon _{1}t +\sum _{r=1}^\infty \sum _{j=0}^l h_{rj}\dfrac{t^{r\beta +j}}{\varGamma (r\beta +j+1)}. \end{aligned}$$
  Next, let $\vartheta _{0(s,l)}(t)$ denote the *sth* truncated series of $\vartheta _{0}(t)$ which take the form:
  33 $$\begin{aligned} \vartheta _{0(s,l)}(t)=\varUpsilon _{0}+\varUpsilon _{1}t +\sum _{r=1}^s\sum _{j=0}^l h_{rj}\dfrac{t^{r\beta +j}}{\varGamma (r\beta +j+1)}, \forall s=1,2,... \ and\ l=0,1, \end{aligned}$$
  where $\varUpsilon _{0}$ and $\varUpsilon _{1}$ can be obtained by solving Eqs. (22) and (27). The RPSA assumes the solution of Eqs. (30) and (31) using FPS at $t_0=0$ as:
  34 $$\begin{aligned} {\left\{ \begin{array}{ll} \begin{aligned} \vartheta _{0}(t)=\varPsi _{0}+\varPsi _{1}t +\sum _{r=1}^\infty \sum _{j=0}^l f_{rj}\dfrac{t^{r\beta +j}}{\varGamma (r\beta +j+1)},\\ \vartheta _{1}(t)=\eta _{0}+\eta _{1}t +\sum _{r=1}^\infty \sum _{j=0}^l d_{rj}\dfrac{t^{r\beta +j}}{\varGamma (r\beta +j+1)}. \end{aligned} \end{array}\right. } \end{aligned}$$
  Let $\vartheta _{0(s,l)}(t)$ and $\vartheta _{1(s,l)}(t)$ denote the *sth* truncated series of $\vartheta _{0}(t)$ and $\vartheta _{1}(t)$ which take the form:
  35 $$\begin{aligned} {\left\{ \begin{array}{ll} \begin{aligned} \vartheta _{0(s,l)}(t)=\varPsi _{0}+\varPsi _{1}t +\sum _{r=1}^s\sum _{j=0}^l f_{rj}\dfrac{t^{r\beta +j}}{\varGamma (r\beta +j+1)},\\ \vartheta _{1(s,l)}(t)=\eta _{0}+\eta _{1}t +\sum _{r=1}^s\sum _{j=0}^l d_{rj}\dfrac{t^{r\beta +j}}{\varGamma (r\beta +j+1)},\\ \forall s=1,2,\ldots \ and\ l=0,1, \end{aligned} \end{array}\right. } \end{aligned}$$
  where $\varPsi _{0},$ $\varPsi _{1},$ $\eta _{0}$ and $\eta _{1}$ can be obtained by solving Eqs. (28) and (22). We can write the residual functions of Eqs. (29)–(31) as:
  36 $$\left\{ {\begin{array}{*{20}l} {\Re {\mathfrak{e}\mathfrak{s}}_{{(s,l)}} (t) = {\mathfrak{D}}_{t}^{\beta } \vartheta _{{0(s,l)}} (t) - \frac{1}{8}{\mathfrak{D}}_{t}^{\beta } (A_{1} (t) + A_{2} (t)) - 4\mu (A_{1} (t) + A_{2} (t))} \hfill \\ {\quad + 8\mu \vartheta _{{0(s,l)}} (t) - \frac{3}{4}{\mathbb{L}}(\frac{4}{3}\;\vartheta _{{0(s,l)}} - \frac{1}{6}[A_{1} (t) + A_{2} (t)]),} \hfill \\ \end{array} } \right.$$
  37 $$\left\{ {\begin{array}{*{20}l} {\Re {\mathfrak{e}\mathfrak{s}1}_{{(s,l)}} (t) = {\mathfrak{D}}_{t}^{\beta } \vartheta _{{0(s,l)}} (t) - \frac{{16\mu }}{3}[A_{1} (t) + A_{2} (t)] + \frac{{32\mu }}{3}\vartheta _{{0(s,l)}} (t)} \hfill \\ {\quad - \frac{1}{2}[{\mathbb{L}}(\vartheta _{{0(s,l)}} (t) - \frac{3}{2}\vartheta _{{1(s,l)}} (t) + \frac{1}{8}[A_{2} (t) - A_{1} (t)])} \hfill \\ {\quad + {\mathbb{L}}(\vartheta _{{0(s,l)}} (t) + \frac{3}{2}\vartheta _{{1(s,l)}} (t) - \frac{1}{8}[A_{2} (t) - A_{1} (t)])],} \hfill \\ \end{array} } \right.$$
  38 $$\left\{ {\begin{array}{*{20}l} {\Re {\mathfrak{e}\mathfrak{s}2}_{{(s,l)}} (t) = {\mathfrak{D}}_{t}^{\beta } \vartheta _{{1(s,l)}} (t) - \frac{1}{{12}}{\mathfrak{D}}_{t}^{\beta } (A_{2} (t) - A_{1} (t)) - 8\mu [A_{2} (t) - A_{1} (t)]} \hfill \\ {\quad + 32\mu \vartheta _{{1(s,l)}} (t) + \frac{1}{3}[{\mathbb{L}}(\vartheta _{{0(s,l)}} (t) - \frac{3}{2}\vartheta _{{1(s,l)}} (t) + \frac{1}{8}[A_{2} (t) - A_{1} (t)])} \hfill \\ {\quad - {\mathbb{L}}(\vartheta _{{0(s,l)}} (t) + \frac{3}{2}\vartheta _{{1(s,l)}} (t) - \frac{1}{8}[A_{2} (t) - A_{1} (t)])],} \hfill \\ \end{array} } \right.$$
  *and*
  39 $$\left\{ {\begin{array}{*{20}l} {{\mathfrak{D}}_{t}^{{(r - 1)\beta }} {\mathfrak{D}}_{t}^{j} \;\Re {\mathfrak{e}\mathfrak{s}}_{{(s,l)}} (t_{0} ) = 0,} \hfill \\ {{\mathfrak{D}}_{t}^{{(r - 1)\beta }} {\mathfrak{D}}_{t}^{j} \;\Re {\mathfrak{e}\mathfrak{s}1}_{{(s,l)}} (t_{0} ) = 0,} \hfill \\ {{\mathfrak{D}}_{t}^{{(r - 1)\beta }} {\mathfrak{D}}_{t}^{j} \;\Re {\mathfrak{e}\mathfrak{s}2}_{{(s,l)}} (t_{0} ) = 0,} \hfill \\ {\forall r = 1,2, \ldots ,s\;and\;j = 0,1, \ldots ,l.} \hfill \\ \end{array} } \right.$$
- Time fractional pseudo hyperbolic PDEs with nonlocal conditions^34^
  40 $$\begin{aligned} {\mathfrak {D}}^{\beta }_{t} \varTheta (x,t)-\varepsilon \ {\mathfrak {D}}_{t} {\mathfrak {D}}^{2}_{x} \varTheta (x,t)-{\mathfrak {D}}^{2}_{x} \varTheta (x,t)-W(x,t)=0, \ 1<\beta \le 2,\ x\in [0,X],\ t \in [0,T], \end{aligned}$$
  subject to ICs and BCs:
  41 $$\begin{aligned} {\left\{ \begin{array}{ll} \varTheta (x,0)=V_1(x),\ \quad {\mathfrak {D}}_{t} \varTheta (x,0)=V_2(x), x \in [0,X],\\ \varTheta (0,t)= \rho _1 (t)+\displaystyle \int _{0}^{X} \varTheta (x,t)dx = B_1(t),\ t \in [0,T], &{} \\ \varTheta (X,t)=\rho _2 (t)+\displaystyle \int _{0}^{X} \varTheta (x,t)dx=B_2(t), \ t \in [0,T].\\ \end{array}\right. } \end{aligned}$$
  Let us utilize the approximation of $\varTheta _n(x,t)$ which defined in Eq. (19) as following steps:
  Step (I) By substituting Theorem (1) and Eq. (19) into Eq. (40), we obtain
    42 $$\begin{aligned} \sum _{i=0}^n {\mathfrak {D}}^{\beta }_{t} \vartheta _{i}(t){\mathfrak {T}}^{*}_i(x)-\varepsilon \sum _{i=\lceil \beta \rceil }^n \sum _{k=0}^{i-\lceil \beta \rceil } {\mathfrak {D}}_{t}\vartheta _{i}(t) \varOmega _{i,k} ^{(\beta )} x^{i-k-\beta }-\sum _{i=\lceil \beta \rceil }^n \sum _{k=0}^{i-\lceil \beta \rceil } \vartheta _{i}(t) \varOmega _{i,k} ^{(\beta )} x^{i-k-\beta }-W(x,t)=0. \end{aligned}$$
  Step (II) By collocating Eq. (42) at the roots $x_p,$ $p = 0,1,2, \ldots n-\lceil \beta \rceil$ and the collocation point of SCPSK ${}^{*}_{n+1-\lceil \beta \rceil } (x)$, we get a system of FODEs as:
    43 $$\begin{aligned} \sum _{i=0}^n {\mathfrak {D}}^{\beta }_{t} \vartheta _{i}(t){\mathfrak {T}}^{*}_i(x_p)-\varepsilon \sum _{i=\lceil \beta \rceil }^n \sum _{k=0}^{i-\lceil \beta \rceil } {\mathfrak {D}}_{t}\vartheta _{i}(t) \varOmega _{i,k} ^{(\beta )} x_p^{i-k-\beta }-\sum _{i=\lceil \beta \rceil }^n \sum _{k=0}^{i-\lceil \beta \rceil } \vartheta _{i}(t) \varOmega _{i,k} ^{(\beta )} x_p^{i-k-\beta }-W(x_p,t)=0. \end{aligned}$$
  Step (III) By substituting Eq. (19) into Eq. (41), we can obtain $(\lceil \beta \rceil +1)$ algebraic equations as:
    44 $${\left\{ \begin{array}{ll}\sum _{i=0}^n \vartheta _{i}(0){\mathfrak {T}}^{*}_i(x)=V_1(x),\\ \sum _{i=0}^n {\mathfrak {D}}_{t}\vartheta _{i}(0){\mathfrak {T}}^{*}_i(x)=V_2(x),\end{array}\right. }$$
    where BCs
    45 $$\begin{aligned} {\left\{ \begin{array}{ll} \begin{aligned} \sum _{i=0}^n \vartheta _{i}(t){\mathfrak {T}}^{*}_i(0)= B_1(t),\\ \sum _{i=0}^n \vartheta _{i}(t){\mathfrak {T}}^{*}_i(X)=B_2(t). \end{aligned} \end{array}\right. } \end{aligned}$$
    To obtain the unknown coefficients $\vartheta _0(t),\vartheta _1(t),\vartheta _2(t), \ldots ,\vartheta _n(t)$, combing Eqs. (43)–(45), we have system of FODEs which can be solved by utilizing RPSA. To determine the unknown coefficients of $\vartheta _0(t),\vartheta _1(t),\vartheta _2(t), \ldots ,\vartheta _n(t)$, we take $n=3$ in Eq. (43), we have
    46 $$\begin{aligned} {\left\{ \begin{array}{ll} \begin{aligned} {\mathfrak {D}}^{\beta }_{t} \vartheta _{0}(t)-{\mathfrak {D}}^{\beta }_{t} \vartheta _{1}(t)+ {\mathfrak {D}}^{\beta }_{t} \vartheta _{3}(t)-\varepsilon {\mathfrak {D}}_{t}\big (32\vartheta _{2}(t)-96\vartheta _{3}(t)\big )-\big (32\vartheta _{2}(t) -96\vartheta _{3}(t)\big )-W\left(\dfrac{1}{4},t\right)=0,\\ {\mathfrak {D}}^{\beta }_{t} \vartheta _{0}(t)+{\mathfrak {D}}^{\beta }_{t} \vartheta _{1}(t)- {\mathfrak {D}}^{\beta }_{t} \vartheta _{3}(t)-\varepsilon {\mathfrak {D}}_{t}\big (32\vartheta _{2}(t)+96\vartheta _{3}(t)\big )-\big (32\vartheta _{2}(t) +96\vartheta _{3}(t)\big )-W\left(\dfrac{3}{4},t\right)=0. \end{aligned} \end{array}\right. } \end{aligned}$$
    By solving Eq. (45), we get
    47 $$\begin{aligned} {\left\{ \begin{array}{ll} \begin{aligned} \vartheta _{2}(t)=\dfrac{1}{6}\bigg (B_1(t)+B_2(t)\bigg )-\dfrac{1}{3}\vartheta _{0}(t),\\ \vartheta _{3}(t)= \dfrac{1}{8}\bigg (B_2(t)-B_1(t)\bigg )-\dfrac{1}{2}\vartheta _{1}(t).\\ \end{aligned} \end{array}\right. } \end{aligned}$$
    By solving Eq. (46), we obtain
    48 $${\left\{ \begin{array}{ll} {\mathfrak {D}}^{\beta }_{t} \vartheta _{0}(t)-32\varepsilon {\mathfrak {D}}_{t}\vartheta _{2}(t)-32\varepsilon \vartheta _{2}(t)-\dfrac{1}{2}\bigg (W(\dfrac{1}{4},t)+W(\dfrac{3}{4},t)\bigg )=0,\\ {\mathfrak {D}}^{\beta }_{t} \vartheta _{1}(t)- {\mathfrak {D}}^{\beta }_{t} \vartheta _{3}(t)-96\varepsilon {\mathfrak {D}}_{t}\vartheta _{3}(t)-96 \vartheta _{3}(t)+\dfrac{1}{2}\bigg (W(\dfrac{1}{4},t)-W(\dfrac{3}{4},t)\bigg )=0.\end{array}\right. }$$
    By substituting Eq. (47) into Eq. (48), then
    49 $${\left\{ \begin{array}{ll} {\mathfrak {D}}^{\beta }_{t} \vartheta _{0}(t)-\dfrac{16\epsilon }{3}\ {\mathfrak {D}}_{t}\bigg (B_1(t)+B_2(t)\bigg )+\dfrac{32\epsilon }{3}{\mathfrak {D}}_{t}\vartheta _{0}(t)\\ -\dfrac{16}{3}\ \bigg (B_1(t)+B_2(t)\bigg )+\dfrac{32}{3}\vartheta _{0}(t)-\dfrac{1}{2}\bigg (W(\dfrac{1}{4},t) +W(\dfrac{3}{4},t)\bigg )=0,\\\end{array}\right. }$$
    50 $$\begin{aligned} {\left\{ \begin{array}{ll} \begin{aligned} {\mathfrak {D}}^{\beta }_{t} \vartheta _{1}(t)- \dfrac{1}{12}{\mathfrak {D}}^{\beta }_{t} \bigg (B_2(t)-B_1(t)\bigg )-8\varepsilon {\mathfrak {D}}_{t}\bigg (B_2(t)-B_1(t)\bigg )+32\varepsilon {\mathfrak {D}}_{t}\vartheta _{1}(t)\\ -8\bigg (B_2(t)-B_1(t)\bigg )+32\vartheta _{1}(t) +\dfrac{1}{3}\bigg (W(\dfrac{1}{4},t)-W(\dfrac{3}{4},t)\bigg )=0. \end{aligned} \end{array}\right. } \end{aligned}$$
    Let $\vartheta _{0(s,l)}(t)$ and $\vartheta _{1(s,l)}(t)$ denote the *sth* truncated series of $\vartheta _{0}(t)$ and $\vartheta _{1}(t)$ which defined in Eq. (35), then the residual functions of Eqs. (49) and (50) take the form:
    51 $$\begin{aligned} {\left\{ \begin{array}{ll} \begin{aligned} \mathfrak {Res1}_{(s,l)}(t)={\mathfrak {D}}^{\beta }_{t} \vartheta _{0(s,l)}(t)-\dfrac{16\epsilon }{3}\ {\mathfrak {D}}_{t}\bigg (B_1(t)+B_2(t)\bigg )+\dfrac{32\epsilon }{3}{\mathfrak {D}}_{t}\vartheta _{0(s,l)}(t)\\ -\dfrac{16}{3}\ \bigg (B_1(t)+B_2(t)\bigg )+\dfrac{32}{3}\vartheta _{0(s,l)}(t)-\dfrac{1}{2}\bigg (W(\dfrac{1}{4},t) +W(\dfrac{3}{4},t)\bigg ), \end{aligned} \end{array}\right. } \end{aligned}$$
    52 $${\left\{ \begin{array}{ll}\mathfrak {Res2}_{(s,l)}(t)={\mathfrak {D}}^{\beta }_{t} \vartheta _{1(s,l)}(t)- \dfrac{1}{12} {\mathfrak {D}}^{\beta }_{t} \bigg (B_2(t)-B_1(t)\bigg )-8\varepsilon {\mathfrak {D}}_{t}\bigg (B_2(t)-B_1(t)\bigg )\\ +32\varepsilon \ {\mathfrak {D}}_{t}\vartheta _{1(s,l)}(t)-8\bigg (B_2(t)-B_1(t)\bigg )+32\vartheta _{1(s,l)}(t) +\dfrac{1}{3}\bigg (W(\dfrac{1}{4},t)-W(\dfrac{3}{4},t)\bigg ),\end{array}\right. }$$
    *and*
    53 $${\left\{ \begin{array}{ll} {\mathfrak {D}}^{(r-1)\beta }_{t}{\mathfrak {D}}^{j}_{t}\ \mathfrak {Res1}_{(s,l)}(t_0)=0,\\ {\mathfrak {D}}^{(r-1)\beta }_{t}{\mathfrak {D}}^{j}_{t}\ \mathfrak {Res2}_{(s,l)}(t_0)=0, \\ \forall r=1,2, \ldots ,s \ and\ j=0,1, \ldots ,l.\end{array}\right. }$$

## Numerical simulation

Two problems are established in this section to demonstrate the effectiveness and applicability of the CTSCSK-RPSA.

**Problem 1.** Suppose the following nonlinear time fractional hyperbolic PDEs^29^ which are described in Eq. (17), where $\mu =0$ and ${\mathbb {L}}\bigg ( \varTheta (x,t)\bigg )=\dfrac{\partial }{\partial x}\bigg (\varTheta (x,t) \dfrac{\partial \varTheta (x,t)}{\partial x}\bigg )$, then

54 $$\begin{aligned} {\mathfrak {D}}^{\beta }_{t} \varTheta (x,t)- \dfrac{\partial }{\partial x}\bigg (\varTheta (x,t) \dfrac{\partial \varTheta (x,t)}{\partial x}\bigg )=0, \ 1<\beta \le 2,\ x\in [0,1],\ t >0, \end{aligned}$$

with ICs and BCs:

55 $$\begin{aligned} {\left\{ \begin{array}{ll}\varTheta (x,0)=x^2, &{} {\mathfrak {D}}_{t} \varTheta (x,0)=-2x^2,\\ \varTheta (0,t)=0, &{} \varTheta (1,t)=\dfrac{1}{(t+1)^2}. \\ \end{array}\right. } \end{aligned}$$

The exact solution at $\beta =2$ is $\varTheta (x,t)=\dfrac{x^2}{(t+1)^2}.$

**Figure 1:**
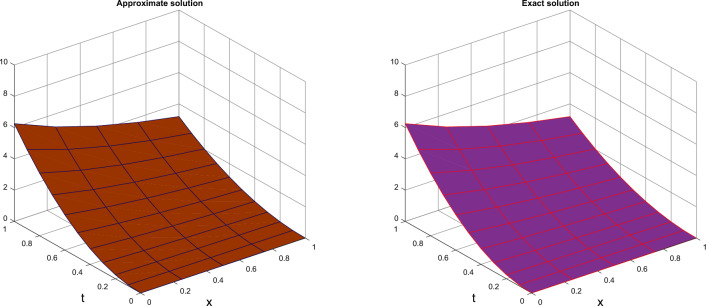
Exact and approximate solutions at $\beta =2$ of Problem 1.

**Figure 2:**
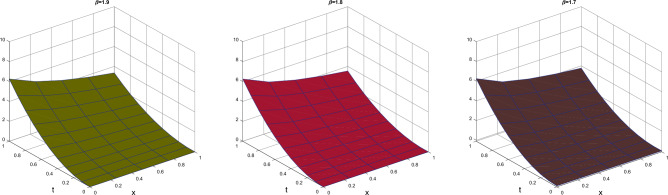
Behavior of approximate at different values of $\beta$ for Problem 1.

**Figure 3:**
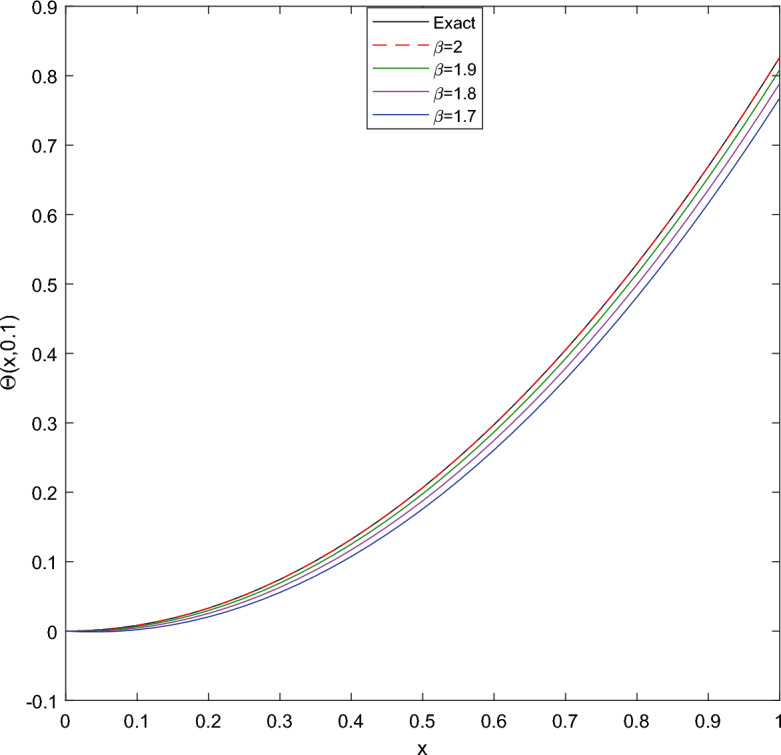
2D graphics of exact and approximate solutions at different fractional order of $\beta$ for Problem 1.

Table 1 presents the approximate solutions obtained by CTSCSK-RPSA with VIM, ADM^8^, VHPIM, HPM^11^ and PIA^29^. Table 2 present the CTSCSK-RPSA approximate solutions at various values of $\beta$. Figure 1 represents comparison between exact and approximate solutions at $\beta =2$. Figure 2 shows the 3D graph of approximate solution at $\beta =\{1.9,1.8,1.7\}$. Figure 3 displays the behavior of approximate solution for fractional order $\beta$ and $t=0.1$ in two dimensional graphs.

**Table 1:** The Comparison between CTSCSK-RPSA and other available methods for Problem 1.

*t*	*x*	Exact	CTSCSK-RPSA		VIM^8^	ADM^8^	HPM^11^	VHPIM^11^	PIA^29^
			$n=2$	$n=3$					
0.2	0.25	0.043403	0.043403	0.043403	0.043400	0.043395	0.043400	0.04320	0.043400
0.2	0.5	0.173611	0.173611	0.173611	0.173600	0.173580	0.173600	0.172820	0.173599
0.2	0.75	0.390625	0.390625	0.390625	0.390600	0.390556	0.390600	0.388844	0.390599
0.2	1	0.694444	0.694444	0.694444	0.694400	0.694321	0.694400	0.691278	0.694399
0.4	0.25	0.031888	0.031887	0.031888	0.031779	0.031567	0.031779	0.029913	0.031779
0.4	0.5	0.127551	0.127551	0.127551	0.127118	0.126268	0.127118	0.119650	0.127118
0.4	0.75	0.286990	0.286988	0.286990	0.286015	0.284103	0.286015	0.269212	0.286015
0.4	1	0.510204	0.510204	0.510204	0.508471	0.505072	0.508471	0.478600	0.508472
0.6	0.25	0.024414	0.024648	0.024444	0.023665	0.022005	0.023665	0.018860	0.023665
0.6	0.5	0.097656	0.097968	0.097460	0.094660	0.088018	0.094660	0.075442	0.094659
0.6	0.75	0.219727	0.219960	0.219403	0.212984	0.198040	0.212984	0.169743	0.212984
0.6	1	0.390625	0.390625	0.390625	0.378638	0.352071	0.378638	0.301766	0.378638

**Table 2:** Numerical results of CTSCSK-RPSA at different values of $\beta$ for Problem 1.

*t*	*x*	$\beta =1.5$				$\beta =1.75$			
		ADM^8^	VIM^8^	$n=2$	$n=3$	ADM^8^	VIM^8^	$n=2$	$n=3$
0.2	0.25	0.0592832	0.047502	0.043791	0.043325	0.0497012	0.043403	0.044368	0.043210
0.2	0.5	0.237133	0.190007	0.174129	0.174129	0.194805	0.184170	0.174898	0.174898
0.2	0.75	0.533549	0.427517	0.391013	0.391480	0.438311	0.414383	0.391590	0.392748
0.2	1	0.948532	0.760029	0.694444	0.694444	0.779220	0.736680	0.694444	0.694444
0.4	0.25	0.0654119	0.041853	0.043745	0.023960	0.037742	0.037742	0.032128	0.031840
0.4	0.5	0.261647	0.167412	0.180405	0.180405	0.174992	0.150968	0.127872	0.174898
0.4	0.75	0.588707	0.376676	0.326630	0.374198	0.393732	0.339679	0.287230	0.287519
0.4	1	1.04659	0.669647	0.510204	0.510204	0.699969	0.603873	0.510204	0.510204
0.6	0.25	0.063177	0.037722	0.220672	0.117212	0.381836	0.031457	0.185133	0.100000
0.6	0.5	0.252710	0.150888	0.359333	0.359333	0.152735	0.125829	0.311949	0.311949
0.6	0.75	0.568598	0.339499	0.318117	0.651493	0.343653	0.283114	0.415984	0.554257
0.6	1	1.01084	0.603553	0.390625	0.390625	0.610938	0.503314	0.390625	0.390625

**Problem 2.** Consider time fractional pseudo hyperbolic PDEs with nonlocal conditions^34^

56 $$\begin{aligned} {\mathfrak {D}}^{\beta }_{t} \varTheta (x,t)-\varepsilon \ {\mathfrak {D}}_{t} {\mathfrak {D}}^{2}_{x} \varTheta (x,t)-{\mathfrak {D}}^{2}_{x} \varTheta (x,t)-e^{t}(x^3-18x)=0, \ 1<\beta \le 2,\ x\in [0,1],\ t \in [0,2], \end{aligned}$$

with ICs and BCs:

57 $$\begin{aligned} {\left\{ \begin{array}{ll} \varTheta (x,0)=x^3,&{} {\mathfrak {D}}_{t} \varTheta (x,0)=x^3,\\ \varTheta (0,t)=\displaystyle \int _{0}^{1} \varTheta (x,t)dx-\frac{1}{4} e^{t},&{} \varTheta (1,t)= \displaystyle \int _{0}^{1} \varTheta (x,t)dx+\frac{3}{4} e^{t}. \\ \end{array}\right. } \end{aligned}$$

The exact solution at $\beta =2$ is $\varTheta (x,t)=x^3 e^{t}.$

**Figure 4:**
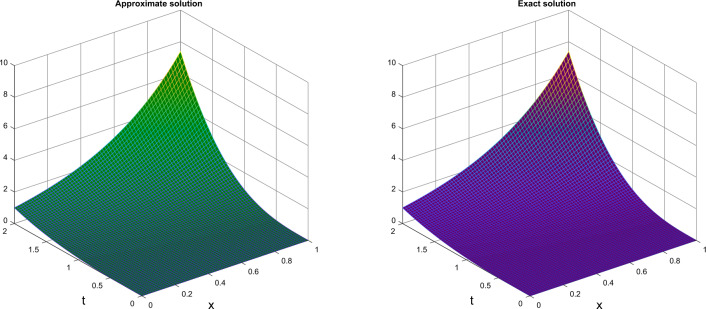
Exact and approximate solutions at $\beta =2$ of Problem 2.

**Figure 5:**
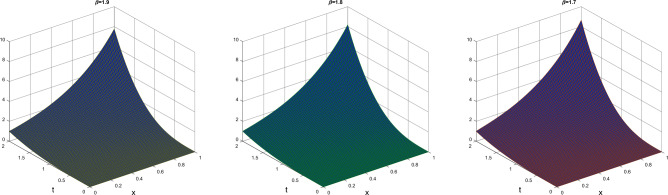
Behavior of approximate at different values of $\beta$ for Problem 2.

**Figure 6:**
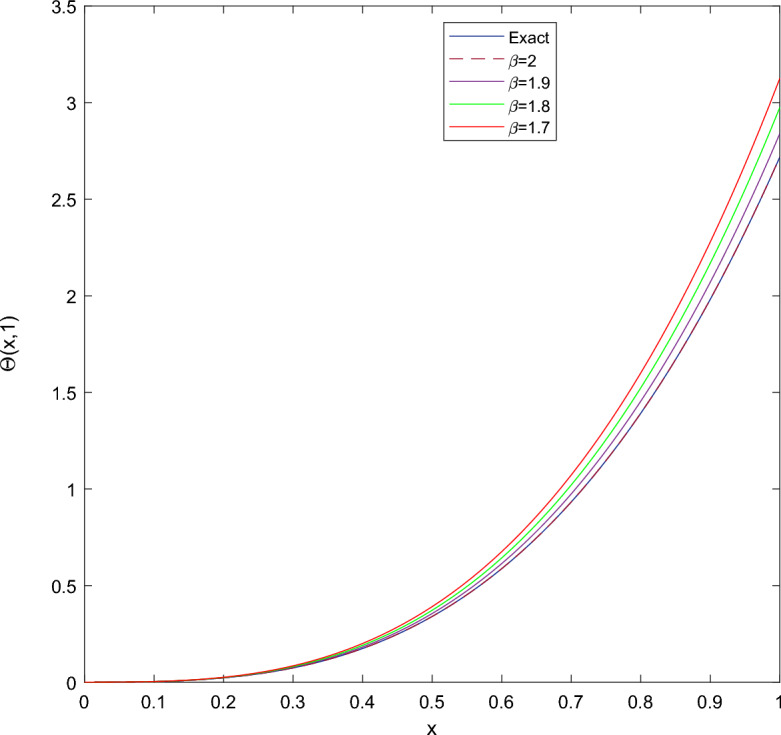
2D graphics of exact and approximate solutions at different fractional order of $\beta$ for Problem 2.

Table 3 shows the numerical solution obtained by CTSCSK-RPSA and RPSA with absolute error. Table 4 present the CTSCSK-RPSA approximate solutions at various values of $\beta$. Figure 4 represents comparison between exact and approximate solutions at $\beta =2$. Figure 5 shows the 3D graph of approximate solution at $\beta = \{1.9,1.8,1.7\}$. Figure 6 displays the behavior of approximate solution for fractional order $\beta$ and $t=1$ in two dimensional graphs.

**Table 3:** Numerical results of pseudo hyperbolic PDE with nonlocal conditions at $\varepsilon =2$ and $\beta =2$ for Problem 2.

*x*	*t*	Exact	Approximate solutions		Absolute error	
			CTSCSK-RPSA	RPSA^34^	CTSCSK-RPSA	RPSA^34^
0	0	0	0	0	0	0
0.1	0.2	0.001221	0.001221	0.001221	$6.49719\times 10^{-14}$	$1\times 10^{-12}$
0.2	0.4	0.011935	0.011935	0.011935	$1.73472\times 10^{-18}$	0
0.3	0.6	0.049197	0.049197	0.049197	$2.77556\times 10^{-17}$	$3\times 10^{-11}$
0.4	0.8	0.142435	0.142435	0.142435	$8.32667\times 10^{-17}$	$1\times 10^{-10}$
0.5	1	0.339785	0.339785	0.339785	0	$3\times 10^{-10}$
0.6	1.2	0.717145	0.717145	0.717145	$1.11022\times 10^{-16}$	0
0.7	1.4	1.390934	1.390934	1.390934	$2.22045\times 10^{-16}$	0
0.8	1.6	3.097420	3.097420	3.097420	$4.44089\times 10^{-16}$	0
0.9	1.8	4.410193	4.410193	4.410193	0	$3\times 10^{-9}$
1	2	7.389056	7.389056	7.389056	$2.66454\times 10^{-15}$	$2.8\times 10^{-8}$

**Table 4:** Approximate solution for different values of $\beta$ for Problem 2.

*x*	*t*	CTSCSK-RPSA		
		$\beta =1.95$	$\beta =1.85$	$\beta =1.75$
0	0	0	0	0
0.1	0.2	0.007560	0.001264	0.001298
0.2	0.4	0.012122	0.012529	0.012985
0.3	0.6	0.050111	0.052087	0.054292
0.4	0.8	0.145352	0.151656	0.158673
0.5	1	0.347200	0.363209	0.381013
0.6	1.2	0.733481	0.768741	0.807946
0.7	1.4	1.423587	1.494070	1.572439
0.8	1.6	2.596795	2.728137	2.874197
0.9	1.8	4.517702	4.749821	5.008006
1	2	7.571329	7.964938	8.402844

## Conclusion

In this study, the CTSCSK-RPSA is successfully applied to solve nonlinear time fractional hyperbolic PDEs and time fractional pseudo hyperbolic PDEs with nonlocal conditions. Error analysis of the proposed problems was studied. It is clear that the numerical and simulation results obtained by CTSCSK-RPSA at $\beta =2$ are close to the exact solutions and they are more accurate than previous methods in the literature. All results were done with MATLAB R2017b (9.3.0.713579). Finally, we point out that CTSCSK-RPSA is a convenient and efficient solutions for for various types of fractional linear and nonlinear problems that arise in engineering and applied physics.

## Data Availability

Data used to support the findings of this study are included in the article.
